# School-type differences in e-cigarette use and its correlates among Chinese adolescents

**DOI:** 10.18332/tid/118721

**Published:** 2020-03-19

**Authors:** Jingfen Zhu, Jiahui Li, Gang Xu, Jinming Yu, Qian Wang, Yaping He

**Affiliations:** 1 College of Public Health, Shanghai Jiao Tong University School of Medicine, Shanghai, China; 2 School of Public Health, Fudan University, Shanghai, China; * Contributed equally; # Co-correspondence authors

**Keywords:** e-cigarettes, adolescents, China, vocational schools

## Abstract

**INTRODUCTION:**

Studies examining e-cigarette use among adolescents in Shanghai, China, have focused largely on middle school students. Given the vast differences between vocational and traditional schools, we aimed to explore school-type differences in e-cigarette use and correlates among adolescents in Shanghai.

**METHODS:**

The study was conducted in September 2017 through multistage and stratified cluster random sampling, which consisted of 10699 adolescents aged 13–17 years attending traditional and vocational schools in Shanghai. Descriptive statistics and multivariate logistic regressions were conducted to assess the weighted prevalence and correlates of ever e-cigarette use stratified by school type.

**RESULTS:**

The weighted prevalence of e-cigarette use was 5.21% among all respondents. Although e-cigarette use was more prevalent among students attending vocational schools (p<0.001), its correlates were similar across both school types. Among vocational school students, ever tobacco use (OR=3.10; 95% CI: 2.36–4.08) was the most significant correlate, followed by having most friends as smokers (OR=2.97; 95% CI: 1.84–4.81) and having morning cravings (OR=1.90; 95% CI: 1.64–2.20). Among traditional school students, having most friends as smokers (OR=4.87; 95% CI: 2.78–8.54) and ever tobacco use (OR=3.78; 95% CI: 2.68–5.34) were the most significant correlates, followed by knowledge of pro-tobacco advertisements (OR=2.12; 95% CI: 1.54–2.91).

**CONCLUSIONS:**

Joint efforts from the national, school and family levels are needed to control e-cigarette use among adolescents in China, and such efforts should be tailored to address differences in school characteristics.

## INTRODUCTION

When e-cigarettes entered the market in the early 2000s, they were often advertised as healthier alternatives to traditional cigarettes or as smoking cessation aids^1^. However, the impact of e-cigarettes on smoking cessation has been found to be inconsistent^2^. Some studies found smokers used e-cigarettes to help them quit smoking^3^, while others found no benefit in using e-cigarettes to quit smoking, or even found a detrimental effect among smokers that used e-cigarettes^4^.

E-cigarette use has increasingly gained popularity among adolescents. A nationally representative survey in the US reported a significant increase in current use of e-cigarettes among middle school (0.6% to 5.3%) and high school students (1.5% to 16.0%) from 2011 to 2015^5^. A British survey found that about a tenth to a fifth of those aged 11–16 years had tried e-cigarettes^6^. In Canada, about 20% of youth aged 15–19 years reported ‘ever tried’ e-cigarettes, of whom 14% were non-smokers^7^. In South Korea, about 9–10% of adolescents were found to be e-cigarette users, of whom 75–76% also used tobacco^8^. The prevalence of e-cigarette use was also high among youth in Taiwan, as a survey showed e-cigarette use was particularly common among those aged 15–24 years who were current (49–52%) or former (22–39%) smokers^9^. In Hong Kong, about 1.1% of secondary school students (mean age=14.8±1.9 years) used e-cigarettes currently, and 11.7 % of e-cigarette users never smoked cigarettes^1^. Even though mainland China is among the world’s largest and major producers of e-cigarettes^10^, and over 90% of the world’s e-cigarettes come from cities such as Shenzhen^1^, studies on e-cigarette use among Chinese adolescents are few. A recent national survey found the prevalence of e-cigarette use and awareness to be 1.2% and 45.0%, respectively, among middle school students in China^11^.

A number of studies have examined factors associated with e-cigarette use among adolescents. E-cigarette use has been strongly linked to smoking and susceptibility to smoking^7^. A study found curiosity was the most commonly cited reason among less frequent e-cigarette users, while the desire to quit smoking and the opportunity for indoor use were the most commonly cited reasons among more frequent users^11^. A national survey conducted among middle school students in China found e-cigarette awareness and use were associated with: cigarette smoking, having parents or close friends who smoke, exposure to pro-tobacco advertising and anti-tobacco messages, having a positive attitude to smoking, and having more pocket money^12^. However, this survey did not include high school students. There is evidence suggesting that e-cigarette use may be more prevalent among high school students. For example, in 2014, the rate of e-cigarette use was 3.9% among middle school students but was 13.4% among high school students in the US^13^. Moreover, in 2015, this rate increased to 5.3% among middle school students and 16.0% among high school students^14^.

In China, compared with traditional high schools, vocational high schools often have low academic requirements. Students attending vocational high schools tend to receive less parental monitoring^15^. These differences may contribute to more health risk behaviors among vocational school students.

The purpose of our study was to explore school-type differences in e-cigarette use and correlates among adolescents in Shanghai. As the largest metropolitan area in China, Shanghai is home to 34 million people. We examined students’ use of e-cigarettes, its associations with tobacco use, morning craving, parents’ and friends’ smoking, exposure to pro- and anti-tobacco advertisements and to secondhand smoking (SHS), with the hope that better policies will be developed for regulating e-cigarette use among youth.

## METHODS

### Sample

This cross-sectional study was conducted in September 2017 through multistage and stratified cluster random sampling. Participants were adolescents attending traditional middle schools and high schools, and also from vocational high schools, in Shanghai. Sixteen districts in Shanghai were stratified into central urban and non-central urban areas, and four districts were randomly selected, with Huangpu and Putuo as central urban areas, and Minhang and Jiading as non-central urban areas. All schools in these four districts were further stratified into middle schools, traditional high schools, and vocational high schools. From these, a total of 33 schools were randomly selected, with one vocational school chosen randomly from each district. A total of 12278 students participated in the study, with 10699 (87.1%) returning questionnaires with all questions used in the analysis completed.

Participation in this study was voluntary. Written informed consent was obtained from all students, their guardians and school organizers before enrollment, and covered objectives, procedures, potential risks and benefits of the study. The study was approved by the Ethics Committee of the School of Public Health, Shanghai Jiao Tong University.

### Measures

Use of electronic cigarettes was the outcome variable of interest and was measured by the question: ‘Have you tried electronic cigarettes (even one puff)?’; with answer options ‘yes/no’.

Morning craving was an independent variable and measured by the question: ‘Do you smoke soon or crave for a cigarette upon waking up in the morning?’; with answer options ‘I don’t smoke’, ‘I haven’t smoked for a long time’, ‘No, I don’t crave for a cigarette in the morning’, ‘Yes, sometimes’, and ‘Yes, often’. This variable was treated as a continuous variable.

Awareness of anti- and pro-tobacco advertisements was assessed separately by asking respondents if they had seen or heard of anti- or pro-tobacco advertisements from four channels in the past 30 days: traditional media (TV, films, broadcasting, newspaper, billboards); digital media (cell phones, computers/laptops, websites); mobile digital billboards on buses or subways; community activities; sports events, concerts, exhibitions, and community gatherings. Response options were: 1) ‘I don’t have the opportunity to see such advertisement’, 2) ‘often (four times and above)’, 3) ‘sometimes (one to three times)’, and 4) ‘No, I haven’t seen such advertisement’. Those who chose 2) and 3) were considered having awareness of anti- or pro-tobacco advertisements, and those choosing 1) and 4) were considered as not having awareness of anti- or pro-tobacco advertisements.

Ever tobacco use was assessed by: ‘Have you ever tried cigarette smoking (even one or two puffs)?’. Participants were grouped into those who answered ‘yes’ versus ‘no’.

Parents’ smoking status was measured by two questions: ‘Does your father smoke?’ and ‘Does your mother smoke?’. Answer options were: 1) ‘No’, 2) ‘Sometimes’, 3) ‘Often’, and 4) ‘I don’t know’. Participants were grouped into three categories based on their responses, those who answered 2) and 3) to both questions were considered as having both parents as smokers, while those who answered 2) and 3) to either one of the two questions were considered as having one parent as a smoker. Those who answered 1) and 4) to both questions were considered as having parents who were non-smokers.

Friends’ smoking status was measured by: ‘Do you have friends that smoke?’. Answer options were: 1) No, 2) Some of them, 3) Majority of them, and 4) All of them; these were then grouped into three categories with 3) and 4) combined.

Secondhand smoke (SHS) was measured by past 7-day exposure to SHS at home, in indoor public places (shops, restaurants, movie theatres, train stations etc.), and at outdoor areas (parks, playgrounds, sidewalks etc.). Response options were: 1) 0 days, 2) 1–2 days, 3) 3–4 days, 4) 5–6 days, and 5) 7 days. Those answering 0 days or 1–2 days to all three sources of exposure to SHS were treated as the group with low exposure, and those answering 3–4 days or above to any of the three sources of exposure to SHS were treated as the group with high exposure.

Demographic covariates included: age (11–13; 14–15; and 16–17 years), gender, residence (local, non-local), grade point average (GPA) (continuous), type of school (middle, high, vocational high school), and monthly allowance (continuous).

### Data analysis

We used Statistical Package for the Social Sciences version 22.0 for all statistical analyses. Data were weighted based on selection probability of districts, the number of schools in each district, and the number of students in each school. Sample characteristics and e-cigarette use characteristics were summarized using weighted percentages and confidence intervals. Odds ratios (ORs) with 95% confidence intervals (CIs) were calculated to examine overall, as well as school-specific associations between each independent variable and e-cigarette use. For each school type, adjusted odds ratios (AORs) with their 95% confidence intervals (CIs) of the associations between e-cigarette use and its correlates were estimated by multivariate logistic regression models, which could account for several confounding variables simultaneously. A p<0.05 was considered statistically significant.

## RESULTS

### Sample characteristics

The overall sample consisted of 10699 students (mean age = 13.8±2.1 years) attending middle school (62.1%), high school (23.9%), or vocational high school (14.0%), in Shanghai (Table 1).

**Table 1:** Characteristics distribution and ever use of e-cigarettes among adolescents, Shanghai, China, September 2017 (N=10669)

*Characteristics*	*Overall*		*Ever use of e-cigarettes*	

	*n*	*Weighted % 95% CI*	*n*	*Weighted % 95% CI*
Overall	10669		695	5.2 (4.8–5.6)
**Age** (years)				
11–13	4751	52.1 (51.1–53.1)	131	2.8 (2.3–3.3)
14–15	3043	25.7 (24.9–26.6)	212	6.0 (5.2–6.9)
16–17	2905	22.2 (21.4–23.0)	352	10.0 (9.0–11.2)
**Gender**				
Female	4968	47.5 (46.5–48.4)	184	3.1 (2.7–3.6)
Male	5731	52.5 (51.6–53.5)	511	7.1 (6.5–7.8)
**Residence**				
Local	7551	71.7 (70.8–72.6)	443	4.8 (4.3–5.2)
Non-local	3148	28.3 (27.4–29.2)	252	6.4 (5.6–7.2)
**Grade point aver age**				
Top 25%	3367	32.5 (31.5–33.4)	200	4.7 (4.1–5.5)
Average	5022	46.7 (45.7–47.7)	254	4.0 (3.5–4.5)
Bottom 25%	2310	20.9 (20.1–21.7)	241	8.7 (7.6–9.8)
**Type of school**				
Middle school	5663	62.1 (61.2–63.0)	181	3.2 (2.8–3.7)
High school	2185	23.9 (23.0–24.8)	116	5.3 (4.4–6.3)
Vocational high school	2851	14.0 (13.5–14.5)	398	14 (12.7–15.3)
**Monthly allowance** (RMB)				
<200	6225	62.1 (61.2–63.0)	227	3.1 (2.7–3.5)
200–399	1895	16.7 (16.0–17.4)	140	6.1 (5.1–7.2)
400–599	1001	8.5 (8.0–9.1)	82	6.1 (4.8–7.6)
600–799	475	3.7 (3.4–4.1)	51	9.1 (6.8–12.0)
800–999	284	2.1 (1.8–2.3)	35	10.9 (7.7–15.2)
≥1000	819	6.9 (6.4–7.4)	160	17.7 (15.2–20.5)
**Morning craving**				
Never	10193	96.2 (95.8–96.5)	435	3.5 (3.2–3.9)
Rarely	149	1.1 (0.9–1.3)	69	45.2 (36.9–53.8)
Sometimes	159	1.1 (0.9–1.3)	81	46.5 (38.4–54.8)
Often	91	0.7 (0.6–0.9)	52	50.6 (39.9–61.4)
Always	107	0.9 (0.8–1.1)	58	52.4 (42.5–62.1)
**Ever tobacco use**				
No	9598	91.6 (91.1–92.1)	341	3.0 (2.7–3.3)
Yes	1101	8.4 (7.9–8.9)	354	29.7 (26.9–32.6)
**Parents’ smoking**				
None	3758	36.0 (35.0–36.9)	176	3.9 (3.4–4.6)
One	6485	59.9 (58.9–60.8)	431	5.1 (4.7–5.7)
Both	456	4.1 (3.8–4.5)	88	17.2 (14.0–21.0)
**Friends’ smoking**				
None	8366	82.3 (81.6–83)	239	2.5 (2.2–2.8)
Some	2039	15.5 (14.8–16.1)	312	13.8 (12.3–15.4)
Most or all	294	2.2 (2.0–2.5)	144	47.3 (41.3–53.4)
**Exposure to tobacco promotional advertisement**				
No	4063	39.1 (38.1–40)	170	3.2 (2.7–3.7)
Yes	6636	60.9 (60.0–61.9)	525	6.5 (6.0–7.1)
**Exposur e to tobacco contr ol advertisement**				
No	979	8.8 (8.2–9.3)	94	8.7 (7.1–10.7)
Yes	9720	91.2 (90.7–91.8)	601	4.9 (4.5–5.3)
**Secondhand smoke**				
Low exposure	6118	57.4 (56.4–58.4)	241	3.0 (2.6–3.4)
High exposure	4581	42.6 (41.6–43.6)	454	8.2 (7.4–9.0)

RMB: Chinese renminbi, 100 RMB about 14 US$. CI: confidence interval.

The total prevalence of e-cigarette use was 5.2%. Prevalence was significantly higher among adolescents aged 16–17 years (10.1%), males (7.1%), non-local residents (6.4%), attending vocational high schools (14.0%), had ever tried using tobacco (29.7%), had both parents (17.2%) or most friends (47.3%) as smokers, had seen pro-tobacco advertisements (6.5%) or not seen anti-tobacco advertisements (8.7%), and were highly exposed to SHS (8.2%).

### Logistic regressions

In the unadjusted model (Table 2), the associations between ever use of e-cigarettes and all correlates were significant (p<0.001). In the adjusted model (Table 3), correlates of e-cigarette use were assessed stratified by school type. For each school type, e-cigarette users were compared against non-users (reference group) on morning craving, parents’ and friends’ smoking status, knowledge of pro-tobacco advertisements, ever use of tobacco products, and exposure to SHS, controlling for age, gender, GPA, and monthly allowance.

**Table 2:** Correlates of ever e-cigarette use among adolescents stratified by type of school, Shanghai, China, September 2017 (N=10669)

	*Overall Crude OR (95% CI)^d^*	*Vocational Crude OR (9 5% CI)^d^*	*Traditional Crude OR (9 5% CI)^d^*
**Type of school**			
Vocational vs Traditional	4.13 (3.53–4.82)^c^	N/A	N/A
**Age** (years)			
11–13 vs 16–17	0.25 (0.21–0.31)^c^	N/A	0.42 (0.32–0.56)^c^
14–15 vs 16–17	0.57 (0.47–0.69)^c^	0.56 (0.45–0.70)^c^	0.72 (0.53–0.99)^a^
**Gender**			
Male vs Female	2.41 (2.00–2.89)^c^	2.58 (2.02–3.29)^c^	2.13 ( 1.66–2.73)^c^
**Residence**			
Non-local vs Local	1.36 (1.15–1.62)^c^	1.22 (0.98–1.51)	1.24 (0.97–1.59)
**Grade point aver age** (cont.)	1.40 (1.24–1.59)^c^	1.18 (1.01–1.39)^a^	1.41 (1.18–1.68)^c^
**Monthly allowance** (cont.)	1.44 (1.38–1.51)^c^	1.23 (1.16–1.30)^c^	1.45 (1.36–1.54)^c^
**Morning craving** (cont.)	2.92 (2.64–3.23)^c^	2.91 (2.49–3.41)^c^	2.78 (2.47–3.12)^c^
**Parents’ smoking**			
One vs None	1.32 (1.09–1.60)^c^	1.60 (1.24–2.06)^c^	1.08 (0.84–1.40)^c^
Both vs None	5.05 (3.77–6.77)^c^	3.85 (2.53–5.84)^c^	5.25 (3.62–7.62)^c^
**Friends’ smoking**			
Some vs None	6.37 (5.28–7.67)^c^	3.37 (2.63–4.32)^c^	5.93 (4.55–7.73)^c^
Most or all vs None	35.66 (26.98–47.14)^c^	16.36 (11.4–23.47)^c^	37.74 (25.74–55.34)^c^
**Exposure to tobacco promotional advertisement**			
Yes vs No	2.12 (1.76–2.57)^c^	1.39 (1.10–1.76)^b^	2.38 (1.81–3.13)^c^
**Exposure to tobacco control advertisement**			
Yes vs No	0.54 (0.42–0.68)^c^	1.03 (0.74–1.43)	0.43 (0.32–0.59)^c^
**Ever tobacco use**			
Yes vs No	13.78 (11.55–16.46)^c^	7.52 (6.01–9.40)^c^	13.96 (10.85–17.94)^c^
**Secondhand smoke**			
High exposure vs Low exposure	2.87 (2.41–3.41)^c^	2.34 (1.89–2.90)^c^	3.27 (2.55–4.20)^c^

CI: confidence interval. OR: odds ratio. a p<0.05, b p<0.01, c p<0.001. d Data were weighted.

**Table 3:** Correlates of ever e-cigarette use among adolescents stratified by type of school, Shanghai, China, September 2017 (N=10669)

	*Overall AOR (95% CI)^d^*	*Vocational AOR (95% CI)^d^*	*Traditional AOR (95% CI)^d^*
**Type of school**			
Vocational vs Traditional	1.66 (1.32–2.10)^c^	N/A	N/A
**Age** (years)			
11–13 vs 16–17	0.77 (0.58–1.04)	N/A	0.86 (0.61–1.21)
14–15 vs 16–17	0.97 (0.77–1.22)	0.78 (0.61–1.01)	1.14 (0.78–1.65)
**Gender**			
Male vs Female	1.91 (1.54–2.37)^c^	1.78 (1.33–2.36)^c^	1.99 (1.49–2.65)^c^
**Residence**			
Non-local vs Local	1.01 (0.81–1.26)	0.92 (0.70–1.19)	1.07 (0.79–1.45)
Grade point average (cont.)	1.19 (1.04–1.37)^a^	1.09 (0.92–1.30)	1.24 (1.03–1.50)^a^
Monthly allowance (cont.)	1.14 (1.08–1.2)^c^	1.11 (1.03–1.18)^b^	1.13 (1.05–1.23)^b^
Morning Craving (cont.)	1.68 (1.50–1.88)^c^	1.90 (1.64–2.20)^c^	1.58 (1.36–1.83)^c^
**Parents’ smoking**			
One vs None	0.90 (0.72–1.13)	1.29 (0.96–1.74)	0.77 (0.57–1.03)
Both vs None	1.72 (1.15–2.59)^b^	1.73 (1.01–2.98)^a^	1.68 (1.01–2.80)^a^
**Friends’ smoking**			
Some vs None	2.31 (1.82–2.95)^b^	1.68 (1.25–2.24)^b^	2.72 (1.97–3.75)^c^
Most or all vs None	4.14 (2.78–6.16)^c^	2.97 (1.84–4.81)^c^	4.87 (2.78–8.54)^c^
**Exposur e to tobacco promotional advertisement**			
Yes vs No	1.86 (1.48–2.35)^c^	1.42 (1.05–1.91)^a^	2.12 (1.54–2.91)^c^
**Exposur e to tobacco contr ol advertisement**			
Yes vs No	0.79 (0.57–1.11)	0.91 (0.58–1.42)	0.76 (0.49–1.18)
**Ever tobacco u se**			
Yes vs No	3.50 (2.75–4.45)^c^	3.10 (2.36–4.08)^c^	3.78 (2.68–5.34)^c^
**Secondhand smoke**			
High exposure vs Low exposure	1.68 (1.36–2.07)^c^	1.27 (0.98–1.64)	1.93 (1.44–2.59)^c^

CI: confidence interval. AOR: adjusted odds ratio. a p<0.05, b p<0.01, c p<0.001. d Data were weighted.

Among students attending vocational schools, ever use of tobacco was the most significant correlate, with ever users having 3.10 (95% CI: 2.36–4.08) times increased odds of e-cigarette use. Friends’ smoking was also a significant correlate, with those reporting most or all of their friends as smokers having 2.97 (95% CI: 1.84–4.81) times increased odds of e-cigarette use, and those reporting some friends as smokers having 1.68 (95% CI: 1.25–2.24) times increased odds of e-cigarette use. Odds of e-cigarette use were 1.78 (95% CI: 1.33–2.36) times higher than for males, 1.73 (95% CI: 1.01–2.98) times higher for students with both smoking parents, and 1.42 (95% CI: 1.05–1.91) times higher for those who had knowledge of pro-tobacco advertisements. For each unit increase in morning craving and monthly allowance, odds of e-cigarette use increased by 1.90 (95% CI: 1.64–2.20) and 1.11 (95% CI: 1.03–1.18) times, respectively.

Among students attending traditional schools, friends’ smoking was the most significant correlate, with those reporting most or all of their friends smoking having 4.87 (95% CI: 2.78–8.54) times increased odds of using e-cigarettes, and those reporting some friends as smokers having 2.72 (95% CI: 1.97–3.75) times increased odds of using e-cigarettes. Ever use of tobacco was also a significant correlate, with ever users having 3.78 (95% CI: 2.68–5.34) times increased odds of e-cigarette use. Odds of e-cigarette use were 2.12 (95% CI: 1.54–2.91) times higher for those who had seen pro-tobacco advertisements, 1.99 (95%CI: 1.49–2.65) times greater for males, 1.93 (95% CI: 1.44–2.59) times greater for students with high exposure to SHS, and 1.68 (95% CI: 1.01–2.80) times higher for those reporting both parents as smokers. In addition, for each unit increase in morning craving, monthly allowance, or GPA, odds of e-cigarette use increased by 1.58 (95% CI: 1.36–1.83) times, 1.13 (95% CI: 1.05–1.23) times, and 1.24 (95% CI: 1.03–1.50) times, respectively.

## DISCUSSION

To our knowledge, this study is one of a limited number examining school-type differences in correlates of e-cigarette use among adolescents in Shanghai, China. We found e-cigarette use more prevalent among students attending vocational rather than traditional schools. However, correlates of e-cigarettes use were similar, regardless of type of school.

Our finding on the higher prevalence of e-cigarette use among students attending vocational schools may be explained by the nature of vocational schools. In China, students at vocational schools often receive less monitoring and discipline from teachers and parents^16^, have significantly lower academic pressure, and enter the workforce directly after graduation. They are more likely to be exposed to social smokers than do students at traditional schools^17^, and their interests can be easily influenced by the so-called ‘being cool’ culture that involves also smoking^18^. These characteristics may also explain our finding that GPA was a significant correlate of e-cigarette use only among students at traditional instead of vocational schools. More specifically, at traditional schools, students with lower GPAs had higher odds of using e-cigarettes. Another study, among Finnish adolescents, also found that poor school performance was associated with e-cigarette experimentation^19^. The possible reason could be that those students who do not perform well academically suffer more academic burdens and intense peer competition. Taken together, this evidence implies a need for school-specific e-cigarette control policies. Traditional schools may need to pay more attention to underachieving students and help them reduce stress. Vocational schools may need to invest in helping students to build resilience as a means of avoiding the temptations of e-cigarettes.

Our finding that, regardless of school type, the association between tobacco use and e-cigarette use, as well as morning craving, is consistent with existing findings that e-cigarette use was associated with morning smoking urge^20^ and ever tobacco use^11,20^. No longitudinal study has explored the causal relationship between tobacco and e-cigarette use^7^. Our finding implies that e-cigarette control programs should also include elements targeting tobacco use simultaneously. However, because we did not find a significant association between anti-tobacco advertisements and e-cigarette use, advertisements aimed at curbing the use of tobacco may not be an effective strategy.

Our finding that pro-tobacco advertisements increased odds of e-cigarettes use by 1.72 times, is similar to a study that found exposure to tobacco product advertisements raised e-cigarette awareness^11^. This suggests that those who are aware of or use e-cigarettes may be more attentive to or have greater exposure to tobacco-related messages. Tobacco promotion advertisements may market e-cigarettes as socially attractive, with celebrity endorsement and stylish design, and availability in a wide range of flavors^21^. There is a need to regulate tobacco promotion advertising and promotion concerning e-cigarette marketing.

We also found exposure to SHS increased odds of e-cigarette use among traditional school students, but this correlation was of borderline significance among vocational school students. The newly revised *Regulations of Shanghai Municipality on smoking control in public places* came into effect on 1 March 2017 and bans smoking in all indoor areas^22^. In spite of this, SHS exposure still remains a serious problem, as we found 42.6% of respondents had high exposure to SHS overall. Another study reported that the prevalence of passive smoking among traditional middle (67%) and high (63.2%) school students was lower than at vocational schools (75.6%) in Shanghai^23^. Given the risks of exposure to SHS and its influence on e-cigarette use, reducing exposure to SHS requires the joint efforts of the whole of society.

We found odds of e-cigarette use were higher among those having more friends as smokers, and this association was more significant among traditional school students. A study among Finnish adolescents^19^ drew similar conclusions that friends were the main source of e-cigarette initiation. Adolescents tend to consider e-cigarettes as attractive, less harmful, and less addictive if their friends are users^8^. Schools are thus recommended to make full use of peer education to disseminate accurate information on e-cigarette use and encourage positive learning. Similarly, consistent with existing studies^1^, we found adolescents whose parents were both smokers had twice the odds of using e-cigarettes. Parents’ smoking can have a subtle influence on teenagers^24^; it is possible that living with people who smoke can increase awareness and use of e-cigarettes^25^. Therefore, parents are encouraged to create a smoke-free environment at home.

There was also a positive correlation between monthly allowance and odds of e-cigarette use. This finding is consistent with a study among Canadian youth that found having more pocket money was positively associated with e-cigarette use^26^. Pocket money was also associated with cigarette smoking among teenagers^27^. Adolescents with higher monthly allowance have extra money to spend and, thus, can afford e-cigarettes^8^. There is a need for parents and educators to limit and monitor teens’ use of their monthly allowance.

### Strengths and limitations

Our study has several limitations. First, due to the cross-sectional design, causal inference cannot be drawn. Longitudinal studies are needed to examine causal relationships between e-cigarette use and its correlates. Second, our study only surveyed adolescents in Shanghai, so our results may not represent all Chinese adolescents. Nonetheless, our study is one of the earliest studies examining this topic. Third, our study only analyzed ever use of e-cigarettes due to a low prevalence rate of current use, thus the degree of severity of e-cigarette use might not be fully captured.

Despite these limitations, our study has important implications for e-cigarette control policies and research. There is a need for joint efforts at the national, school and family level to reduce e-cigarette use among adolescents. At the national level, a study in 2016 identified 68 countries that had existing or new regulations on e-cigarettes; common regulations included restrictions on age-of-purchase, on sales and marketing, and bans on vaping in public places^28^. By introducing rigorous regulations on e-cigarettes, the United Kingdom has kept its use among those aged 11–16 years at 3% or less^6^. Canada has placed strict regulations on nicotine-containing e-cigarettes^26^, leading to little advertising or marketing in the traditional media^7^. Experience in these countries suggests that implementing regulations on e-cigarettes could be quite effective. We can raise the minimum age to purchase e-cigarettes and other tobacco products to 21 years^29^, and levy excise tax on e-cigarettes. We could also restrict the sales of e-cigarettes^9^, whether containing nicotine or not, and prohibit e-cigarettes from being marketed as smoking cessation aids or sold in a form resembling tobacco products. At the school level, since school is the main place where adolescents gather and receive education, educators play a vital role in guiding adolescents to participate in health promotion activities. In the past decades, China has been committed to carrying out school-based tobacco control programs. But when it comes to e-cigarette use, there is rarely any school-based prevention or intervention program^1^. For example, despite the fact that many e-cigarettes contain the addictive substance nicotine, advertisers have long been promoting e-cigarettes as tobacco-free and harmless, which can lead to increased perceptions of attractiveness among uninformed adolescents. Therefore, adolescents need to be taught how to critically process information contained in advertising. Smoking prevention should not be limited to implementing smoking bans, providing adolescents with knowledge about the impact of smoking on health is also critical, and school is a very good place for such teaching activities. At the family level, parents are responsible for ensuring a smoke-free environment and limiting their teens’ monthly allowance.

## CONCLUSIONS

The use of e-cigarettes among students in traditional schools is lower than in vocational schools. However, correlates of e-cigarettes use were similar regardless of type of school, including poor academic performance, ever tobacco use, morning craving, tobacco promotion advertisement, exposure to secondhand smoke, having friends or parents who smoke, and higher monthly allowance. Reducing e-cigarette use among adolescents requires the joint efforts of schools, families and the whole of society.
